# Acute or chronic periprosthetic joint infection? Using the ESR ∕ CRP ratio to aid in determining the acuity of periprosthetic joint infections

**DOI:** 10.5194/jbji-6-229-2021

**Published:** 2021-06-08

**Authors:** Zachary K. Christopher, Kade S. McQuivey, David G. Deckey, Jack Haglin, Mark J. Spangehl, Joshua S. Bingham

**Affiliations:** Department of Orthopaedic Surgery, Mayo Clinic Arizona, Phoenix, AZ, USA

## Abstract

**Introduction**: The gold standard for determining the duration of periprosthetic joint infection (PJI) is a thorough history. Currently, there are no well-defined objective criteria to determine the duration of PJI, and little evidence exists regarding the ratio between ESR (mm/h) and CRP (mg/L) in joint arthroplasty. This study suggests the ESR / CRP ratio will help differentiate acute from chronic PJI. **Methods**: Retrospective review of patients with PJI was performed. Inclusion criteria: patients >18 years old who underwent surgical revision for PJI and had documented ESR and CRP values. Subjects were divided into two groups: PJI for greater (chronic) or less than (acute) 4 weeks and the ESR / CRP ratio was compared between them. Receiver-operating characteristic (ROC) curves were evaluated to determine the utility of the ESR / CRP ratio in characterizing the duration of PJI. **Results**: 147 patients were included in the study (81 acute and 66 chronic). The mean ESR / CRP ratio in acute patients was 0.48 compared to 2.87 in chronic patients (p<0.001). The ESR / CRP ROC curve demonstrated an excellent area under the curve (AUC) of 0.899. The ideal cutoff value was 0.96 for ESR / CRP to predict a chronic (>0.96) vs. acute (<0.96) PJI. The sensitivity at this value was 0.74 (95 % CI 0.62–0.83) and the specificity was 0.90 (95 % CI 0.81–0.94). **Conclusions**: The ESR / CRP ratio may help determine the duration of PJI in uncertain cases. This metric may give arthroplasty surgeons more confidence in defining the duration of the PJI and therefore aid in treatment selection.

## 1 Introduction

Periprosthetic joint infections (PJIs) affect only 1 %–2 % of all primary total joint arthroplasties but are associated with significant cost and morbidity (Kapadia et al., 2016). As the number of total joint arthroplasties each year continues to rise, so too will the number of PJIs (Dale et al., 2009; Yokoe et al., 2013). Several factors contribute to the successful treatment of PJI – of which infection duration may be one of the most important (Mortazavi et al., 2011; Hoell et al., 2016; Pignatti et al., 2010; Supreeth et al., 2020; Kim et al., 2020). The exact cutoff of an “early” PJI remains controversial, ranging from 4 weeks after index procedure to 90 d. However, there is evidence that treatment with debridement and implant retention is more effective when management occurs within 4 weeks of the index event (Argenson et al., 2019). Using this commonly accepted definition, acute PJIs (aPJIs) present within 4 weeks of the index procedure (≤4 weeks) and are believed to be the result of intraoperative seeding of implants or a hematogenous spread in the early post-operative period (Kapadia et al., 2016; Zimmerli et al., 2004). Acute PJIs may also be the result of hematogenous spread in a previously well-functioning joint arthroplasty with a symptom duration of ≤4 weeks. Chronic periprosthetic infections (cPJIs) are defined as occurring more than 1 month after the index procedure and may be the result of a low-virulence organism seeded intraoperatively or failed treatment of an acute post-operative infection. Chronic PJIs also include missed hematogenous infections with symptom onset >4 weeks (Kapadia et al., 2016; Zimmerli et al., 2004; Huotari et al., 2015).

The treatments for aPJI and cPJI differ greatly and are typically based largely on infection duration. Debridement and implant retention (DAIR) procedures are commonly used in patients with an aPJI. DAIR procedures offer low morbidity but are less effective in the setting of cPJIs. (Triantafyllopoulos et al., 2015; Koyonos et al., 2011; Azzam et al., 2010). Two-stage exchange arthroplasty is the gold standard for cPJIs, with higher success rates compared to DAIR, but these procedures are often associated with significant morbidity and iatrogenic bone loss (Berend et al., 2013). This can be problematic in revision arthroplasty where bone stock may already be limited and explantation may jeopardize the limb. Unfortunately, the delineation between aPJI and cPJI can be difficult to ascertain as oftentimes patients are unable to provide an accurate time frame of symptom onset and duration. This uncertainty can complicate the treatment algorithm.

Diagnosing PJI involves an extensive workup. Inflammatory markers are a first line investigation (Parvizi and Gehrke, 2014). The erythrocyte sedimentation rate (ESR) and C-reactive protein (CRP) are two well-studied inflammatory markers that have been validated to assist in the diagnosis of PJI (Parvizi and Gehrke, 2014; Parvizi et al., 2011, 2018; Bingham et al., 2020). Although often used simultaneously as indicators of inflammation, these markers independently serve as indicators of inflammation acuity. CRP is an acute-phase reactant produced by hepatocytes and secreted into blood plasma at concentrations that are proportionate to bodily inflammation (Pepys and Hirschfield, 2003). Secondary to its shorter half-life when compared to ESR, CRP is more representative of acute inflammatory processes. Conversely, ESR is a marker of chronic inflammation. In proinflammatory states there is an increase in the production of fibrinogen in blood which causes red blood cells (RBCs) to stick together in stacks called rouleaux (Bray et al., 2016). As its name suggests, ESR is the rate at which RBCs in anticoagulated blood descend in a standardized test tube over a 1 h period. Roleaux RBCs settle more quickly than individual RBCs secondary to their increased density, leading to increased ESR values (Tishkowski and Gupta, 2020).

ESR and CRP have significant utility outside the field of orthopedics. Recently, rheumatologists have used the ratio of ESR to CRP to distinguish acute inflammation from flares in chronic inflammatory diseases. One such example would be a patient with lupus who presents to the hospital with new onset fevers in the setting of suspected new infection (Littlejohn et al., 2018). The ESR / CRP ratio could be used to distinguish a fever in the setting of a lupus flare (chronic inflammatory condition) vs. fever secondary to an acute infectious process (acute inflammation). This application of the ESR / CRP ratio demonstrates utility in distinguishing between acute vs. chronic inflammatory states. Therefore, we postulate that the ESR / CRP ratio could aid in determining the chronicity of a PJI. We hypothesize that the ESR / CRP ratio will be significantly lower in aPJIs compared to cPJIs. Utilizing the ESR / CRP ratio in the setting of PJI could help determine the chronicity of infection and guide treatment.

## 2 Methods

### 2.1 Data collection

This study was determined to be minimal risk and approved by the institutional review board. A retrospective chart review was performed to identify all patients diagnosed with a PJI who underwent surgical management between 2000 and 2016. In all patients, the diagnosis of a PJI was confirmed retroactively based on the revised 2014 Musculoskeletal Infection Society (MSIS) criteria (Parvizi and Gehrke, 2014). These criteria included at least one major criterion (sinus tract communicating with prosthesis, two positive periprosthetic cultures with the same pathogen collected on separate occasions) or three minor criteria (elevated ESR and CRP, elevated synovial leukocyte count or ++ change on a leukocyte esterase test strip, elevated synovial neutrophil percentage, positive histologic analysis of periprosthetic tissue, or a single positive culture). Patients were included in the study if they (1) met the 2014 MSIS criteria for PJI, (2) had ESR and CRP labs recorded within 15 d prior to treatment, (3) underwent surgical management for PJI and (4) had clear determination of symptom duration noted in the chart.

For each patient, the duration of infection was determined based on the history of symptom onset provided during their clinical consultation. Acute PJI was defined as those patients with infection within 4 weeks of the previous surgery or less than or equal to 4 weeks of symptoms (acute hematogenous). Symptoms included pain, swelling, erythema, fevers, chills, or onset of a recent inciting infection. Chronic PJI was defined as symptoms of greater than 4 weeks' duration or 4 weeks after the index procedure. To minimize reporting errors and unreliable patient histories, patients were only included in the study if the date or time frame of symptom onset was specifically and clearly documented in the medical record. If a specific time frame according to the operating surgeon was not listed, the patient was excluded. Using very stringent criteria, only historically accurate documentation was included to provide maximum confidence in the timelines. All patients in the acute category were not only diagnosed, but were also treated operatively within the 4-week cutoff period. If they were diagnosed in the acute period but surgery was after 4 weeks, these patients were excluded. Patients were also excluded if they did not have ESR and CRP values within 15 d prior to surgery for both acute and chronic groups. Importantly, all patients included in this study were confirmed to have surgery within the 4-week window from symptom onset if in the acute group. Patient charts were reviewed retrospectively, and data were collected on demographics, diagnosis, lab values (including ESR and CRP), surgical procedure, culture data, ASA score, and the presence of a concomitant inflammatory disease.

### 2.2 Data analysis

The patients were separated into two groups: acute and chronic based on timing of symptom onset. The groups were analyzed to determine any differences in ASA score, age, gender, or underlying preexisting inflammatory diseases. ESR / CRP ratios were calculated for each patient. CRP was measured in mg/L and ESR was measured in mm/h. The data were examined, and summary statistics were computed. T tests, Chi-square tests and Mann–Whitney U tests were used to compare categorical and continuous data that were parametric and non-parametric. A receiver-operating characteristic (ROC) curve was created to assess the diagnostic ability of the ESR / CRP ratio in determining the acuity of an infection. Youden's statistic was utilized to define the optimal cutoff value of the ESR / CRP (JMP^®^, Version 15.2.1. SAS Institute Inc., Cary, NC).

## 3 Results

Two hundred and eighty patients were identified in the study time frame. Ninety-five patients were excluded for failure to meet the diagnostic criteria for a PJI due to insufficient data, while an additional 41 patients were excluded because they lacked either ESR or CRP within 15 d prior to surgery or the timing of their infection was not clearly documented in the medical record with a specific date or time of onset. Ultimately, 146 patients were included in the study. Based on patient history, 81 presented as an aPJI (≤4 weeks) and 65 presented as a cPJI (>4 weeks). There were no differences between these groups with regards to age, gender, ASA score, or the presence of a comorbid inflammatory disease (Table 1). The mean ESR / CRP ratio in the acute cohort was 0.48 compared to 2.87 in the chronic cohort (p<0.001).

**Table 1:** Summary statistics of acute vs. chronic PJI.

	Acute	Chronic	p value
Total number of patients (n)	81	65	
Age			
Mean value (years)	68.46	69.03	0.41
Standard deviation	13.58	8.71	
Gender (n)			
Male	38	33	0.64
Female	43	32	
ASA score			
Mean value	2.84	2.68	0.07
Standard deviation	0.51	0.56	
Inflammatory disease (n)			
Present	14	14	0.52
Absent	67	51	
ESR / CRP ratio			
Mean value	0.48	2.87	<0.001
95 % confidence interval	0.38–0.57	1.78–4.01	

**Figure 1:**
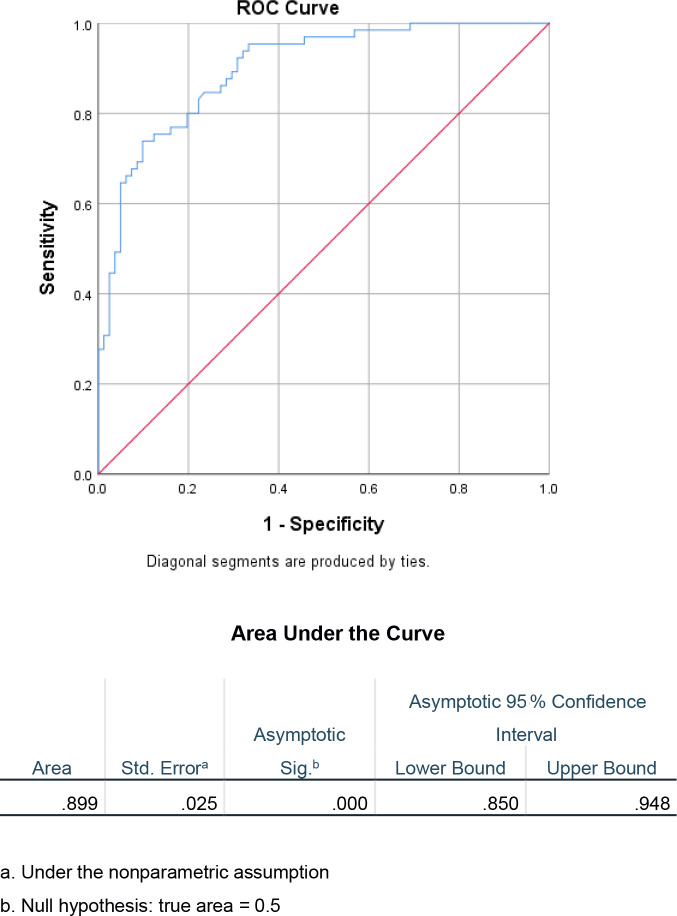
ROC curve and AUC for ESR / CRP ratio in acute vs. chronic PJI.

Receiver-operating characteristic curves were created to evaluate the utility of the ESR / CRP ratio as a diagnostic test in determining the acuity of a PJI. The area under the curve (AUC) was calculated to be 0.899 (Fig. 1). Furthermore, the ideal cutoff value for determining chronicity of infection using the ESR / CRP was then determined. Using Youden's statistic, the optimal cutoff value was approximately 0.96, with a sensitivity of 0.74 and a specificity of 0.90 (Table 2).

**Table 2:** The ideal cutoff values for the ESR / CRP ratio.

	Value of	Sensitivity	Specificity
	ESR / CRP		
	ratio		
Optimal Youden's	0.958	0.738	0.901

## 4 Discussion

PJI is a devastating complication and is the second most common reason for revision joint arthroplasty behind aseptic loosening (Koh et al., 2014). The treatment course is commonly based on the duration of PJI. This presents a challenge for arthroplasty surgeons because it is often difficult to (1) define infection duration based on history alone and (2) to discern the definitions of acute and chronic infections in the literature. For example, a 2018 study proposing a new definition for PJI used only chronic infections to develop its diagnostic scoring model. There was no prior validation that the criteria would apply for acute infections (Parvizi et al., 2018). Other studies have established criteria based on synovial fluid analysis using 6 weeks from the index procedure as the cutoff (Bedair et al., 2011). Are these criteria applicable to acute hematogenous infections that present outside of the post-operative window? Landmark publications such as these are often used as the basis for PJI research, but subsequent studies may differ in their definition of acute or chronic. According to the International Consensus Meeting (ICM) on Musculoskeletal Infection, there is limited evidence to suggest a time interval that would divide acute and chronic PJI (Elkins et al., 2019). They recommend considering multiple factors prior to initiating treatment, including overall patient health, organism virulence, and implant-related factors.

The lack of a consistent method to define acute or chronic PJI makes selecting a treatment challenging. Although there is controversy, current data suggest a benefit in using DAIR procedures for acute infections and a two-stage exchange procedure for chronic infections (Osmon et al., 2013). Although DAIR procedures are recommended in the acute setting, a debate persists given that DAIR procedures have questionable success rates ranging from 35 % to 90 % (Azzam et al., 2010; Ottesen et al., 2019; Deirmengian et al., 2003). In an attempt to improve eradication rates, new intraoperative techniques have been proposed to improve upon the DAIR procedure (Shaw et al., 2017; Chung et al., 2019; Calanna et al., 2019). Although interval improvements have been made to the traditional DAIR, timing remains a critical factor. One guiding principle to success with these procedures is to initiate treatment rapidly after symptom onset. Frequently, patients are unsure of symptom onset or duration and often provide unclear timing of symptom onset history. This lack of detailed history often leads to a difficult decision where the surgeon must determine which procedure will best eradicate the PJI while trying to minimize the morbidity to the patient. We propose that the ESR / CRP ratio could be used as an additional tool to aid in this often-difficult but not uncommon scenario. Once the diagnosis of PJI has been established, this ratio can assist in deciphering the acuity of the infection and guide therapy. As these are values associated with the initial infection workup, there are no additional costs to patients or hospital systems.

Based on the results of this study, the ESR / CRP ratio is a helpful diagnostic test to determine the acuity of a PJI. Receiver-operating curves are useful for predicting how well a model can distinguish between two classes, in this case acute and chronic PJIs (Fan et al., 2006). When evaluating the diagnostic ability of this test with an ROC curve, the AUC was 0.899, which is considered excellent (Mandrekar, 2010). Youden's statistic was chosen, as it has been previously validated to determine the ideal cutoff value for ESR / CRP to correctly identify acute vs. chronic PJI (Unal, 2017). This method maximized the sensitivity and specificity of the test (Youden, 1950). The optimal value was defined as approximately 0.96 with a sensitivity of 0.74 and a specificity of 0.90 (Table 2). This value can be adjusted to increase sensitivity or specificity; however, it was found that using this threshold maintained a high specificity at 0.90 without decreasing sensitivity substantially. In clinical practice rounding the ESR / CRP ratio to 1.0 may be useful to quickly evaluate the duration of a PJI. By rounding the cutoff value to 1, the sensitivity and specificity do not change substantially. If the ESR is greater than the CRP, then it is more likely to be chronic. If the ESR is less than the CRP, it is more likely to be acute. This is a simple way to help assess PJI duration and can add a valuable data point to the PJI equation. One last critical point is to ensure the correct units when calculating the ESR / CRP ratio. In this study, we used mm/h for ESR and mg/L for CRP. Using alternative units (particularly for CRP) will change the resulting ratio by a factor of 10 if CRP is reported in mg/dL.

There are several limitations in this study. First, this is a retrospective study design which has inherent reporting and recall biases. Second, differences in the timing of collecting the ESR and CRP labs could have led to differing values. All labs were collected within 15 d prior to surgery, but it is unclear how variability in timing could affect these results. Third, conditions that affect ESR (such as chronic inflammatory diseases) may alter patients' baseline ESR or CRP levels. However, we attempted to control for differences between groups by identifying these comorbidities. We were able to demonstrate no difference in inflammatory diseases between groups, which should limit the impact of these conditions on the study results. Additionally, as no patients in this study were found to have an adverse local tissue reaction, we do not know whether this ratio applies in patients with an adverse local tissue reaction. Fourth, acute flairs in chronically infected patients would be expected to present with a markedly elevated CRP. This would decrease the ratio and falsely lead to the diagnosis of an acute infection. The ratio does not differentiate between acute infections or patients with chronic infection and acute flairs. However, a thorough history should lead one to suspect a chronic infection. Finally, to determine the duration of infection, we used patient history, which is subject to reporting bias. However, we attempted to minimize reporting errors by only including patients whose symptom duration was clearly reported in the patient records and excluded patients whose documentation was incomplete.

## 5 Conclusion

The ESR / CRP ratio is a useful metric that can be used as an additional tool to help determine the duration of PJI in uncertain cases. Based on these results, an ESR / CRP ratio >1 is suggestive of a chronic PJI, and an ESR / CRP ratio <1 suggests an acute PJI. This metric may help guide treatment by providing arthroplasty surgeons with an additional tool in the setting of uncertain duration of symptoms, thus helping the surgeon direct the appropriate treatment course in a patient diagnosed with PJI.

## Data Availability

The data from this study were generated from our institutional database and are not publicly accessible. This may be available upon reasonable request.

## Code availability

Software code used for statistical analysis is available upon reasonable request.
